# First Age- and Gender-Matched Case-Control Study in Australia Examining the Possible Association between *Toxoplasma gondii* Infection and Type 2 Diabetes Mellitus: The *Busselton* Health Study

**DOI:** 10.1155/2020/3142918

**Published:** 2020-03-24

**Authors:** Aus Molan, Kazunori Nosaka, Michael Hunter, Jinxia Zhang, Xiaoni Meng, Manshu Song, Wei Wang

**Affiliations:** ^1^School of Medical and Health Sciences, Edith Cowan University, Joondalup, WA 6027, Australia; ^2^Busselton Population Medical Research Institute, Busselton, WA 6280, Australia; ^3^School of Population and Global Health, University of Western Australia, Nedlands, WA 6009, Australia; ^4^Key Municipal Laboratory of Clinical Epidemiology, School of Public Health, Capital Medical University, Beijing, China 100069

## Abstract

An emerging field of research is starting to examine the association of infectious pathogens with type 2 diabetes mellitus (T2DM). An understudied parasite of interest is *Toxoplasma gondii.* Globally, very few studies have been conducted to investigate this association. Additionally, very little data exists on the prevalence of *T. gondii* in the general Australian population. Our group sought to determine the prevalence, association, and risk factors between *T. gondii* infection and T2DM from a representative Australian human population. Through a cross-sectional, age- and gender-matched case-control study, 150 subjects with T2DM together with 150 control subjects from the Busselton Health Study cohort were investigated. Sera samples were tested for the presence of anti-*T. gondii* IgG and IgM antibodies using enzyme-linked immunosorbent assays. Survey-derived data were also analyzed to evaluate associated risk factors. The IgG seroprevalence was found to be 62% and 66% for the T2DM and control groups, respectively (OR : 0.84; *p* = 0.471). IgM antibodies were detected in 5% of the T2DM patients and in 10% of the controls (OR = 0.51; *p* = 0.135). There were no significant differences between male and female IgG seroprevalence rates for both groups (OR : 0.88, 0.80; *p* = 0.723). The IgG seropositivity rate increased significantly in T2DM patients aged 45-84 years in comparison to those aged 18-44 years (*p* < 0.05), but this was not observed in the control subjects. No risk factors were associated with *T. gondii* seropositivity in both groups. The first Australian study of its kind found *T. gondii* infection in Western Australia to be highly prevalent. The results also showed that there is no serological evidence of an association between *T. gondii* infection and T2DM in the studied subjects. Australian health authorities should focus on raising awareness of toxoplasma infection and target *T. gondii* transmission control. Further studies are needed to clarify the role of *T. gondii* in T2DM.

## 1. Introduction

Diabetes in Australia has reached an epidemic level with an estimated 6% (1.2 million) of the adult population aged 18 years and over living with the condition [1, 2]. Diabetes is also the sixth leading cause of death in Australia, responsible for 10% of all deaths [3]. The majority (86%) of Australian diabetics have type 2 diabetes mellitus (T2DM) with 172 new cases being diagnosed each day [3]. In fact, diabetes is becoming more common with the rate of prevalence more than doubling from 1.5% to 4.7% between 1989-1990 and 2015-2016 [4]. If the incidence of diabetes continues to grow at the present rates, there will be 2.5-3 million people with diabetes in Australia by 2025 growing to 3.5 million by 2030 [4].

In 2016, 1.6 million deaths were caused by diabetes worldwide [5]. This is mainly due to the increase in the numbers of people with T2DM as a result of the increase in life expectancy, genetic predisposition, physical inactivity, dietary changes, the obesity epidemic, and the decreased mortality rates in diabetic individuals [1]. However, there may also be additional unidentified novel risk factors, such as subclinical inflammation caused by infectious agents, that contribute to this rising prevalence of T2DM [6]. In this regard, an emerging field of research is beginning to investigate the potential of infectious and environmental pathogens to cause low-grade inflammation that may facilitate the risk and development of various metabolic conditions, including diabetes and obesity.

*Toxoplasma gondii* (*T. gondii*) has been identified as pathogen of potential interest in this field. Considered one of the most successful human parasites [7], the Centers for Disease Control and Prevention has prioritised*T. gondii* as one of the top “Five Neglected Parasitic Infections” due to the severity of illness, high incidence, and potential for prevention [8]. Humans acquire *T. gondii* infection by the ingestion of food, water, or soil contaminated by oocysts from the definitive hosts, cats; consumption of raw/undercooked meat and sausages which contains bradyzoites; or vertical transmission of tachyzoites by transfusion, transplantation, or ingestion of raw milk [9, 10]. It has also been hypothesized that *T. gondii* may be transmitted via sexual contact [9]. The global prevalence rates of this parasite are phenomenal with figures ranging from 15 to 85% depending on social habits, climate condition, hygienic standards, and geographical regions [11].

Infection can be present with various nonspecific signs and symptoms, but most are similar to general flu-like indicators [12]. In all infections, specific antibodies to this parasite remain detectable in the serum throughout the life of the host [10]. *Toxoplasma gondii* can infect and replicate in any nucleated host cells, leading to the production of various inflammatory markers via the innate acute inflammatory responses and antigen-specific adaptive immunity. This facilitates a state of chronic inflammation at various anatomical sites in the host [13, 14]. Several reports have linked chronic *T. gondii* infection to several autoimmune disorders such as thyroid disease, systemic sclerosis, rheumatoid arthritis, and inflammatory bowel syndrome with several studies demonstrating a positive correlation between *T. gondii* infection and numerous neurological disorders and cancers [10–12, 15, 16].

However, *T. gondii* infection in individuals with T2DM has received little recognition, and human studies investigating *T. gondii* infection in T2DM subjects are scarce. Moreover, there is very limited information on the prevalence, incidence, and epidemiology of the disease in the general Australian human population—perhaps because *T. gondii* infection is not a notifiable disease in Australia and most *T. gondii* infections are asymptomatic. The few previous studies have reported prevalence rates amongst pregnant woman throughout Australia between 23 and 35% [17–20]. *Toxoplasma gondii* latent infection has great potential as a novel target for T2DM intervention and may pave a path for a new field of study, “Toxoplasmic Type 2 Diabetes” [21, 22].

The objectives of this study were to (1) investigate the possible serological relationship between *T. gondii* and T2DM and (2) identify risk factors for *T. gondii* infection. We undertook the first age- and gender-matched case-control study in Australia by utilizing sera and cross-sectional data (respiratory and chest conditions, various disease states, anthropometric measurements, and laboratory biochemical and haematological parameters) collected from a community-dwelling cohort of adults attending the 2005-2007 Busselton Health Survey in Western Australia.

## 2. Materials and Methods

The present study describes samples and data collected from the residents of the inner-regional local government electoral boundary of the City of *Busselton,* Western Australia, a centre for farming, vineyards, timber, tourism, and mineral sands industries. A cross-sectional general population health survey (Busselton Health Study, BHS) was conducted between 2005 and 2007 with participants recruited from the compulsory electoral role. This survey was conducted by the Busselton Population Medical Research Institute (BPMRI), a prominent biobank. Details on recruitment and study protocols from this survey are described in Musk et al. [23]. Ethical approval for the current analyses was obtained from the Edith Cowan University Human Research Ethics Committee (Project Number 16090).

### 2.1. Study Design

The design of the present study was a case-control study in which the seroprevalence of *T. gondii* in subjects with T2DM (*n* = 150) was measured and compared to age- and gender-matched controls (non-T2DM, *n* = 150). Clinical and demographic parameters were also investigated for possible risk factor association. Sera were analyzed for the presence of IgG and IgM antibodies against *T. gondii* using commercially available qualitative ELISA methods (Demeditec Diagnostics GmbH, Germany). These *in-vitro* diagnostic assays have been designed for the qualitative determination of specific IgG and IgM antibodies against *T. gondii* in serum and plasma, and have claimed clinical sensitivities and specificities of 99% and 98%, respectively, for the IgG assay, and 99% and 100%, respectively, for the IgM assay.

### 2.2. Criteria for Selection of Participants

Participants with T2DM were defined as cases. T2DM was classified according to the 1999 World Health Organization (WHO) Criteria (fasting plasma glucose greater than or equal to 7.0 mmol/L and/or 2-hour plasma glucose greater than or equal to 11.1 mmol/L) [24]. Healthy participants were defined as controls by reference to the following criteria: (1) did not have a documented medical diagnosis of diabetes, (2) were not taking any glucose-lowering medications, and (3) have fasting and 2-hour glucose values below the diagnostic thresholds for diabetes. In addition, subjects were screened for history of treatment with psychoactive medication and were excluded because of the previously reported and established associations between positive *T. gondii* serology and numerous forms of mental illnesses [15, 16].

### 2.3. Sample and Data Collection

The present study utilized 20 *μ*L of serum aliquoted from banked samples (stored at -80°C) that have been previously collected from study participants aged 18-80 years attending the 2005-2007 BHS. During this survey, each participant completed a standard self-administered questionnaire that obtained information on respiratory and chest conditions and various disease states and underwent a range of clinical tests including anthropometric, respiratory, and cardiovascular measurements. Each participant had a blood sample collected from the cubital fossa in 10 mL red top clot, 2 mL purple top EDTA (ethylenediaminetetraacetic acid), and 2 mL grey top FlOx (fluoride/oxalate) vacutainer tubes (BD Biosciences, Franklin Lakes, New Jersey, USA) for laboratory biochemical and haematological analyses which were conducted by PathWest Laboratory Medicine-QEII Medical Centre, Nedlands, Western Australia. The serum for the present study was aliquoted and stored at -80°C until used.

### 2.4. Sample Integrity and Laboratory Analysis

To minimize false positive or false negative results, lipemic, haemolysed, icteric, or turbid (bacterially contaminated) samples were excluded. Test samples were diluted 1 : 101 with ready-to-use sample diluent (5 *μ*L serum + 500 *μ*L sample diluent). The total volume of serum required from each subject to perform the IgG and IgM ELISAs was 20 *μ*L (5 *μ*L for IgG ELISA, 5 *μ*L for IgM ELISA, and 10 *μ*L for repeat testing in the event of an equivocal result). The case and control sera were tested for anti-*T. gondii* IgG and anti-*T. gondii* IgM antibodies using the Demeditec Diagnostics DETOX01 (IgG ELISA) and DETOX03 (IgM ELISA) kits according to the manufacturers' instructions. Briefly, these assays have been designed for the qualitative evaluation of specific IgG and IgM antibodies against toxoplasma in serum. The analysis was performed double-blind to avoid result bias. Samples from the female, male, T2DM, and control groups were randomly mixed, and the analyst performing the analysis was not aware of the source of samples. Then, 100 *μ*L of the diluted (1 : 101) sample and the ready-to-use calibrators (IgG [IU/mL]; A, 0; B, 10; C, 40; D, 100; E, 250; IgM [U/mL]; A, 1; B, 10; C, 30; D, 120) were pipetted into each test well (coated with *T. gondii* strain RH antigens, isolated from infected mice, common to both the IgG and IgM assays) leaving one well empty for the substrate blank. The plate was covered and incubated for 60 minutes at room temperature. The wells were then washed three times with 300 *μ*L of diluted washing solution using a Bio-Plex Pro II Microplate Wash Station (Bio-Rad Laboratories, Berkeley, California). Subsequently, 100 *μ*L of ready-to-use conjugate was added into each well except the substrate blank well. The plate was covered and incubated at room temperature for 30 minutes. This was followed by another washing procedure as outlined above, after which 100 *μ*L of the ready-to-use substrate was pipetted into each well including the substrate blank well. A final incubation phase for 20 minutes at room temperature in the dark was performed before terminating the substrate reaction with the addition of 100 *μ*L of the ready-to-use stop solution into each well. The plate was then mixed and the wiped in preparation for reading. This was performed using a FLUOstar Omega microplate reader (BMG Labtech, Offenburg, Germany) at an absorption of 450 nm. A standard curve was generated by plotting the mean absorbance at 450 nm for each standard concentration (*x*-axis) against the target antibody concentration (*y*-axis).

### 2.5. Qualitative Serology Interpretation

Results were determined qualitatively by comparing the calculated absorptions for each sample serum with the value for the cutoff calibrators (IgG, 10 IU/mL; IgM, 10 U/mL). Values higher than the cutoffs (IgG, 10 IU/mL; IgM, 10 U/mL) were considered positive, values less than the cutoffs were considered negative, while values falling within a grayzone of ±20% of the cutoff values were considered equivocal and retested once.

### 2.6. Statistical Analyses

Statistical calculations justifying the sample sizes were performed using G∗Power V3.1.9.2 (Heinrich Heine University Düsseldorf, Germany). The following values were used: power of 95%; a 1 : 1 proportion of cases and controls; and a reference seroprevalence of 50% for the T2DM group and 30% for controls. Thus, a minimum sample size of 139 cases and 139 controls was obtained and rounded up to 150 subjects per group. Basic descriptive statistics including counts, means, standard deviations, and percentages were calculated for the control and T2DM groups and their respective gender- and age- subgroups. Age was divided into seven age groups: 18–34, 35–44, 45–54, 55–64, 65–74, 75–84, and 85 years and older. Seroprevalence rates (%) of *T. gondii* including the odds ratios (OR) and corresponding 95% confidence intervals (95% CI) were calculated. The Fisher's exact test was used to evaluate the seroprevalence values between case and control subjects with respect to categorical variables to determine possible risk factor associations. For continuous quantitative variables (laboratory and anthropometric data), the normality of the data distribution was assessed using the Shapiro-Wilk test, Q-Q (quantile-quantile) plots, and skewness and kurtosis values. As the laboratory and anthropometric data were normally distributed, the parametric independent sample Student's *t-*test was used to compare the selected parameters between the study groups. Correlation was assessed using the Pearson correlation coefficient with *p* value and heat maps was generated to display the interrelationships between the laboratory and anthropometric data within each study group. Probability values were calculated on the basis of two-tailed tests. For all analyses, a *p* value less than 0.05 was considered statistically significant. Data were analyzed using the Statistical Package for Social Sciences (IBM® SPSS® Statistics version 25.0, Armonk, New York, USA).

## 3. Results

In this study, the case (*n* = 150) and control (*n* = 150) cohorts were matched for age, gender, and age group distribution. The mean age of the T2DM and control groups was 59.2 ± 15.1 and 58.4 ± 15.7 years, respectively. The mean duration of T2DM was 6.8 years. Most of patients were in the 55–64 and 65-74 (*n* = 64) age groups, followed by the 45-54 (*n* = 62), 35-44, and 75-84 (*n* = 42) age groups. The basic descriptive and biometric statistics are summarized in Table 1. As expected, T2DM subjects had significantly higher weight, waist, and BMI measurements when compared to the controls. The remainder of the baseline characteristics of the study groups were not significantly different (*p* > 0.05).

### 3.1. *Toxoplasma gondii*, T2DM, and Associated Factors

From the 150 subjects with T2DM, 93 (62.0%) were found to be seropositive for anti-*T. gondii* IgG antibodies in comparison to 99 (66.0%) of the 150 healthy control subjects. The difference between the two groups was not significant (OR : 0.84; *p* = 0.470). Anti-*T. gondii* IgM antibodies were found in 8 (5.0%) of the T2DM patients and in 15 (10.0%) of the control subjects. Again, the difference was not significant between the two groups (OR = 0.51; *p* = 0.129) (Table 2).

Regarding the impact of gender, our results showed similar seroprevalence rates of *T. gondii* IgG antibodies between the female and male subjects belonging to the T2DM and control groups. However, there was a significant difference in the seroprevalence rate of *T. gondii* IgM antibodies between the female T2DM group compared with the female controls (4.0% and 17.3%, respectively; OR : 0.20; *p* = 0.008). This was not observed within the male groups (Table 2).

**Figure 1:**
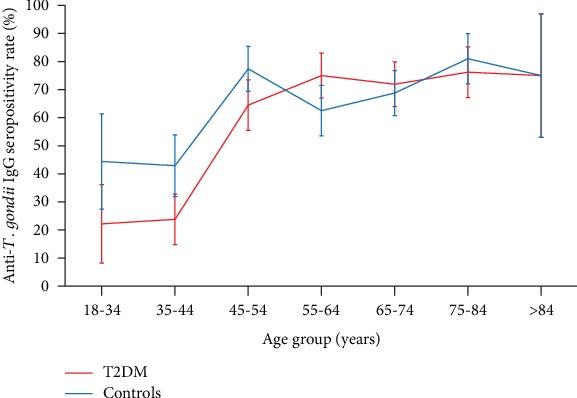
Seroprevalence of anti-*T. gondii* IgG antibodies among patients with T2DM and control subjects, stratified by age group. BHS, Western Australia. Average rate of seropositivity increase was calculated at 0.97% and 0.57%, per year of age, for the T2DM and control groups, respectively. Error bars represent standard error of the respective proportions.

Anti-*T. gondii* IgG seropositivity increased with age at the rates of 0.97% and 0.57%, per year of age, for the T2DM and control groups, respectively (Figure 1). In the T2DM group, the 75-84 age group had the highest seropositivity rate at 76.2% (*p* = 0.011), followed by the 55-64 age group (75.0%; *p* = 0.009), the 65-74 age group (71.9%; *p* = 0.014), and the 45-54 age group (64.5%; *p* = 0.037), whilst the lowest seropositivity of 22.2% was observed in the 18-34 age group. In contrast, in the control subjects, no significant relationship between increasing age and increasing percentage of seropositivity for anti-*T. gondii* IgG antibodies was observed. IgM was exuded from this and further analyses due to the low number of seropositive subjects.

A positive correlation was found between the duration of T2DM and rate of *T. gondii* infection in that T2DM subjects infected with *T. gondii* had been living with T2DM for longer (average of 7.6 years, *p* = 0.044) and were older (average of 63.2 years old, *p* < 0.001) than their non-*T. gondii*-infected T2DM counterparts whom would have been living with T2DM for a shorter period (average of 5.6 years) and were younger (average of 52.8 years old). Likewise, for the control group, a similar trend was observed with infected subjects having a higher average age than the uninfected subjects (average of 60.9 and 54.5, respectively, *p* = 0.019) (Table 3).

Regarding pet ownership as a risk factor associated with *T. gondii* infection, univariate analysis showed that owning a cat, dog, and/or other pet was not significantly associated with *T. gondii* infection. However, subjects with T2DM were less likely than their matched controls to own a cat (OR = 0.57, *p* = 0.140) or other pet (OR = 0.87, *p* = 0.721) while the controls reported a higher rate of dog ownership (OR = 1.32, *p* = 0.344) when compared to the T2DM subjects. In addition, univariate analysis showed that body mass index (BMI) among the selected disease states and lifestyle factors (asthma, arthritis, bronchitis, cancer, eczema, food allergies, hay fever, other chest conditions, pleurisy, pneumonia, and sinusitis) were not identified as risk factors associated with *T. gondii* infection (Table 2).

### 3.2. Laboratory and Anthropometric Analysis

Findings from the laboratory and anthropometric studies are summarized in Table 4. As expected, weight, BMI, and waist circumference measurements were significantly higher in subjects with T2DM in comparison to the control subjects (*p* < 0.01). Results from laboratory investigations showed that elevated white cell count (*p* = 0.016), lymphocyte count (*p* = 0.020), neutrophil count (*p* = 0.039), glucose (*p* < 0.01), insulin (*p* < 0.01), and triglyceride (*p* < 0.01) levels were significantly associated with T2DM. However, T2DM subjects had lower cholesterol (*p* = 0.033) and high-density lipoprotein (*p* < 0.01) results when compared to their control counterparts.

When adjusted for *T. gondii* IgG seropositivity, it was found that seropositive T2DM subjects had a significantly lower red cell distribution width (RDW), a measure of the variation in the size and volume of a patients red blood cells, than seronegative individuals (average of 3.5 and 6.8, respectively; *p* < 0.001). A similar trend was seen in the control group where seropositive subjects had significantly lower RDW values than seronegative individuals (average of 3.1 and 5.8, respectively; *p* < 0.01). Monocyte counts were also lower in seropositive T2DM subjects (*p* = 0.044) while higher neutrophil counts were observed in seropositive control subjects (*p* = 0.032). The remaining anthropometric examinations and laboratory results were comparable between the study groups and no significant association with *T. gondii* seropositivity was found (*p* > 0.05).

**Figure 2:**
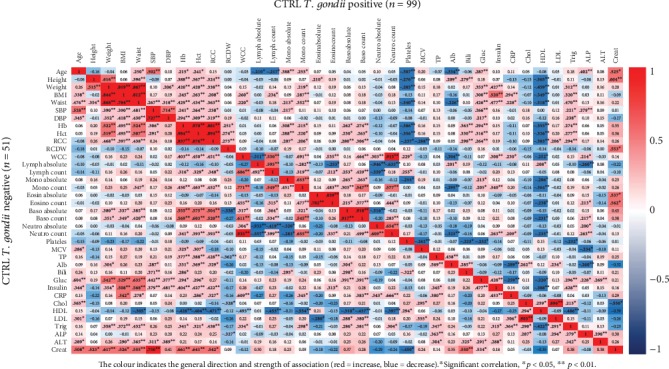
Correlation heat map with Pearson correlation coefficient values displaying the interrelationships between 37 laboratory and anthropometric parameters in control subjects with and without *T. gondii* infection. BHS, Western Australia.

**Figure 3:**
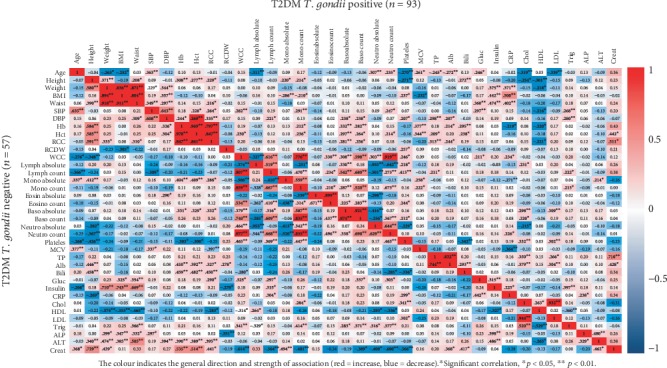
Correlation heat map with Pearson correlation coefficient values displaying the interrelationships between 37 laboratory and anthropometric parameters in T2DM subjects with and without *T. gondii* infection. BHS, Western Australia.

Correlation heat maps were generated to examine possible relationships between *T. gondii* seropositivity and the laboratory and anthropometric determinations in the control and T2DM study groups (Figures 2 and 3, respectively). Moderate positive correlations were observed between creatinine and weight (*r* = 0.62, *p* < 0.01), body mass index (BMI, *r* = 0.53, *p* < 0.01), waist circumference (*r* = 0.56, *p* < 0.01), systolic blood pressure (SBP, *r* = 0.75, *p* < 0.01), hemoglobin (Hb, *r* = 0.66, *p* < 0.01), hematocrit (Hct, *r* = 0.64, *p* < 0.01), red cell count (RCC, *r* = 0.54, *p* < 0.05), and bilirubin (*r* = 0.55, *p* < 0.01) in the control seronegative group but not in the control seropositive group. However, in the control seropositive group, creatinine correlated with red cell distribution width (RCDW, *r* = 0.55, *p* < 0.05) which was not the case for the seropositive group (*r* = 0.09).

Similarly, moderate positive relationships were noted between creatinine and height (*r* = 0.73, *p* < 0.01), Hb (*r* = 0.54, *p* < 0.05), and Hct (*r* = 0.51, *p* < 0.01) in the T2DM seronegative group but not in the seropositive group. However, a strong positive association between creatinine and total protein was evident in the T2DM seropositive group (*r* = 0.71, *p* < 0.01) but not in the seronegative group (*r* = 0.20). All other parameters displayed correlations of negligible magnitude between the *T. gondii* seropositive and seronegative groups in the T2DM and control cohorts.

## 4. Discussion

Globally, few studies have been conducted to explore the association between *T. gondii* infection and diabetes with conflicting reported results [25–32]. Based on the findings of the present study, *T. gondii* infection appears very common in both T2DM patients and healthy non-T2DM control subjects living in Busselton, Western Australia. Our investigation showed that 62.0% of the subjects with T2DM and 66.0% of the healthy control subjects were seropositive for anti-*T. gondii* IgG antibodies with no significant difference observed between the two groups (*p* = 0.84). Therefore, the findings of the present study do not support an association between T2DM and *T. gondii* infection. Similarly, an Iranian descriptive case-control study in which 150 diabetic patients and 150 healthy individuals were tested for anti-*T. gondii* IgG antibodies using an ELISA method found that 52.6% of diabetic patients were IgG seropositive compared to 50.6% of the healthy individuals [25]. In addition, a recent age- and gender-matched case-control study conducted in Mexico also found no serological evidence of an association between *T. gondii* infection and diabetes mellitus [26]. The authors reported low IgG seroprevalence rates of 6.4% (10/156) and 3.2% (5/150) in the diabetic and healthy groups, respectively (OR : 2.06; 95%CI : 0.69 − 6.19; *p* = 0.18).

In contrast, studies that support an association between *T. gondii* infection and diabetes all report significantly higher seroprevalence rates of *T. gondii* infection in subjects with diabetes when compared to the apparently healthy nondiabetic individuals. It should be noted that all the investigations originate from the Middle East and the majority include both T1DM and T2DM patients in the case groups therefore preventing direct comparisons to be made with the present study: Molan et al., Iraq (diabetic, 300/450, 66.6%; control, 68/203, 33.4%; *p* = 0.009) [27], Hemida, Iraq (diabetic, 96/172, 55.8%; control, 38/98, 38.8%; *p* < 0.01) [28]; Saki et al., Iran (diabetic, 47/110, 42.7%; control, 24/110, 21.8%; *p* < 0.05) [29]; Shirabazou et al., Iran (diabetic, 55/91, 60.4%; control, 36/93, 38.0%; *p* < 0.001) [30]; Hemida et al., Egypt (T2DM, 14/37, 37.8%; control, 12/50, 24%; *p* = 0.04) [31], and Gokce et al., Turkey (T2DM, 457/807, 56.6%; control, 56/250, 22.4%; *p* < 0.001) [32].

Recent systematic reviews and meta-analyses [22, 33, 34] conducted to determine the possible association between *T. gondii* infection and diabetes mellitus concluded that *T. gondii* is a possible risk factor for diabetes and that further investigation is recommended. However, the studies included in these meta-analyses share common fundamental weaknesses, especially if they are to be considered reference baseline studies. Firstly, there is a lack of standardisation with regard to the criteria used to define various parameters, especially the diagnosis of diabetes. This includes defining inclusion and exclusion criteria consistent with the latest definitions of diabetes by the WHO. Secondly, there is no mention of having excluded individuals with psychiatric conditions, including personality disorders, from these studies (clinical heterogeneity). Thirdly, the studies were not comparable in their methods of measuring *T. gondii* exposure, a fundamental factor that increases methodical heterogeneity. Lastly and perhaps most importantly, there is noteworthy geographical and demographic skew in favor of the Middle East. To this extent, it is interesting to note that the only two studies outside of the Middle East, the present study and that of Alvarado-Esquivel et al. [26] conducted in Mexico, both found that no significant association between *T. gondii* infection and diabetes.

In the present study, domestic ownership of cats, dogs, or other pets was not identified to be a risk factor associated with *T. gondii* infection. Previous epidemiological studies have reported similar observations [35, 36]. Although the lack of an association of cat ownership may be surprising, due to cats being biologically essential to the life cycle of *T. gondii* as the only definitive hosts, contact with cats appears to be a less important risk factor when compared to other well-established risk factors such as contact with contaminated foods [25]. While data from other studies support cat contact as a risk factor [37–39], prevention of *T. gondii* infection via cat exposure may be possible as cats only shed oocysts for 1-3 periods in a lifetime. In addition, oocyst sporulation can be avoided by regular removal of cat litter [40]. Hence, the results from the current study may indicate that Australian cat owners are looking after their cats very well with regard to their hygiene.

Although some studies have identified some factors such as BMI and some diseases like cancer as risk factors associated with the infection with *T. gondii* [10, 25, 29, 39–41], the results of the present study did not find any association between the selected respiratory and chest conditions, various disease states, or anthropometric measurements and *T. gondii* infection in Western Australia. With respect to the biochemical and haematological laboratory analyses, all parameters except red cell distribution width (RDW) were comparable, and no significant association with *T. gondii* infection was found among the T2DM and control groups. Significantly higher RDW values were found in *T. gondii* seronegative subjects from both the T2DM and control groups when compared to seropositive subjects (*p* < 0.01). The RDW forms part of the standard laboratory full blood count measuring the range of variation in red blood cell volume and recently, higher values have been associated with various disease states including various forms of cancer [42], carotid artery atherosclerosis [43], and metabolic syndrome [44]. Moreover, Patel et al. [45] conducted a mortality follow-up study of 8,175 adults whose RDW values had been previously recorded. They found that higher RDW were strongly associated with an increased risk of death, and RDW is a strong predictor of mortality. Further investigation is warranted to confirm our findings and to explore the causes of lower RDW values from subjects infected with *T. gondii*.

The strengths of the present study include a systematic health screening process leading to a representative study population including appropriate adjustment for gender and age. Lastly, the associated factors were in majority environmental factors hence specific factors like lifetime cat exposure or lifetime undercooked meat consumption, that were not available from the survey, should be investigated in the future.

## 5. Conclusions

We conclude that *T. gondii* infection in Western Australia is highly prevalent. There is no serological evidence of an association between *T. gondii* infection and T2DM in the studied subjects in *Busselton*, Western Australia. Pet ownership, amongst other parameters, was not identified as a risk factor associated with *T. gondii* infection in T2DM and healthy subjects. Toxoplasmosis has been neglected in Australian notifiable disease programs; therefore, public health authorities should focus on raising awareness of *Toxoplasma* infection and introduce public health programs targeting *T. gondii* transmission control. Further studies are needed to clarify the role of *T. gondii* in T2DM.

## Figures and Tables

**Table 1:** Basic descriptive and biometric statistics for the study cohort comparing the case (T2DM subjects) and control (non-T2DM healthy subjects) groups at baseline including age group stratification. BHS, Western Australia.

Parameter	T2DM (*n* = 150)	Control (*n* = 150)	*p*	T2DM M (*n* = 75)	Control M (*n* = 75)	*p*	T2DM F (*n* = 75)	Control F (*n* = 75)	*p*
Average age (years)	59.2 ± 15.1	58.7 ± 15.8	N.S	63.9 ± 13.5	63.5 ± 14.5	N.S	54.6 ± 15.2	54.0 ± 15.8	N.S
Height (cm)	168.1 ± 9.1	168.4 ± 9.3	N.S	174.5 ± 6.7	173.9 ± 7.7	N.S	161.8 ± 6.5	163.0 ± 7.5	N.S
Weight (kg)	85.0 ± 18.5	77.2 ± 15.6	^∗∗^	90.0 ± 14.4	85.8 ± 13.8	^∗∗^	80.1 ± 20.5	68.6 ± 12.2	^∗∗^
Waist (cm)	99.7 ± 14.8	91.3 ± 13.2	^∗∗^	104.6 ± 11.2	99.5 ± 10.2	^∗∗^	96.2 ± 16.7	83.3 ± 10.7	^∗∗^
BMI (kg/m^2^)	30.0 ± 6.0	27.1 ± 4.5	^∗∗^	29.5 ± 4.1	28.4 ± 3.8	^∗∗^	30.5 ± 7.4	25.9 ± 4.9	^∗∗^
Diabetes duration (years)	6.8 ± 5.8	—	—	6.4 ± 4.6	—	—	7.2 ± 6.8	—	

Values are shown as mean ± standard deviation. M: male subjects; F: female subjects; *n*: number of subjects; cm: centimeter; kg: kilogram; BMI: body mass index; kg/m^2^: kilogram force per square meter. Level of significance as estimated by the independent samples Student's *t*-test: N.S: not significant; ^∗^*p* < 0.05; ^∗∗^*p* < 0.01.

**Table 2:** Univariate analysis of the variables associated with the seroprevalence of anti-*T. gondii* IgG antibodies among T2DM patients and control subjects. BHS, Western Australia.

	Toxoplasma serology				OR	95% CI	*p* value
	T2DM (*n*, %)		Controls (*n*, %)				
	POS	NEG	POS	NEG			
*Gender* (*IgG*)							
Male	51 (68.0)	24	53 (70.7)	22	0.88	0.44-1.77	0.723
Female	42 (56.0)	33	46 (61.3)	29	0.80	0.42-1.54	0.507
Total	**93 (62)**	**57**	**99 (66)**	**51**	**0.84**	**0.52-1.35**	**0.470**
*Gender* (*IgM*)							
Male	5 (6.7)	70	2 (2.7)	73	2.61	0.49-13.88	0.246
Female	3 (4.0)	72	13 (17.3)	62	0.20	0.05-0.73	0.008
Total	**8 (5)**	**142**	**15 (10)**	**145**	**0.51**	**0.21-1.23**	**0.129**
*Health*							
SOB walking	19	74	11	88	2.05	0.92-4.59	0.076
Chronic cough	15	78	20	79	0.76	0.36-1.59	0.465
Chronic phlegm	16	77	15	84	1.16	0.54-2.51	0.699
Rhinitis	51	42	46	53	1.40	0.79-2.47	0.275
Wheeze	23	70	21	78	1.22	0.62-2.39	0.562
Chest tightness	27	66	19	80	1.72	0.88-3.37	0.110
*Disease state*							
Asthma	20	73	20	79	1.08	0.54-2.17	0.824
Arthritis	42	51	36	63	1.44	0.81-2.57	0.215
Bronchitis	17	76	22	77	0.78	0.39-1.59	0.507
Cancer	4	89	4	95	1.07	0.26-4.40	0.938
Eczema	12	81	10	89	1.32	0.54-3.22	0.542
Food allergies	12	81	8	91	1.69	0.66-4.33	0.274
Hay fever	28	65	26	73	1.21	0.64-2.27	0.554
Other chest	12	81	10	89	1.32	0.54-3.22	0.542
Pleurisy	8	85	7	92	1.24	0.43-3.56	0.693
Pneumonia	17	76	18	81	1.01	0.48-2.10	0.996
Sinusitis	21	72	19	80	1.23	0.61-2.47	0.563
*Pet ownership*							
Cat ownership	13	80	22	77	0.57	0.27-1.21	0.140
Dog ownership	41	52	37	62	1.32	0.74-2.35	0.344
Other pet ownership	16	77	19	80	0.87	0.42-1.82	0.721
*Body mass index* (*BMI*, *kg/m^2^*)							
Underweight: <18.5	0	0	1	0	**—**	**—**	**—**
Normal: 18.5-24.9	11	10	30	20	0.73	0.26-2.05	0.553
Overweight: 25.0-29.9	40	22	44	16	0.66	0.66-1.43	0.293
Obese: >30.0	42	24	24	15	1.09	0.48-2.48	0.830
*Waist*							
M > 102 cm, F > 88 cm	53	32	33	16	0.8	0.38-1.68	0.561

*n*: number of subjects; POS: number of subjects in which anti-IgG antibodies were detected; NEG: number of subjects in which anti-IgG antibodies were not detected; OR: odds ratio; 95% CI: 95% confidence interval; SOB: shortness of breath; M: male; F: female.

**Table 3:** Duration of diabetes in T2DM subjects with and without *T. gondii* infection as determined by IgG serology. Average age of T2DM and control subjects with and without *T. gondii* infection as determined by IgG serology. BHS, Western Australia.

	Toxoplasma IgG serology in T2DM subjects		*p* value
	POS (*n* = 93)	NEG (*n* = 57)	
Duration of T2DM (years)	7.6 ± 6.2	5.6 ± 4.8	0.044
Age of subjects (years)	63.2 ± 13.0	52.8 ± 16.1	<0.001

	Toxoplasma IgG serology in control subjects	
	POS (*n* = 99)	NEG (*n* = 51)	
Age of subjects (years)	60.9 ± 15.0	54.5 ± 16.7	0.019

Values are shown as mean ± standard deviation. POS: number of subjects in which anti-IgG antibodies were detected; NEG: number of subjects in which anti-IgG antibodies were not detected.

**Table 4:** Comparison of selected laboratory and anthropometric examinations, raw, and after adjustment for *T. gondii* infection status between the T2DM (*n* = 150) and control groups (*n* = 150). BHS, Western Australia.

Parameter	T2DM	Control	*p*	T2DM		*p*	Control		*p*
				IgG POS	IgG NEG		IgG POS	IgG NEG	
Height (cm)	168.1 ± 9.1	168.4 ± 9.3	0.787	167.7 ± 9.4	168.9 ± 8.8	0.455	167.8 ± 9.8	169.7 ± 8.2	0.234
Weight (kg)	85.0 ± 18.4	77.2 ± 15.6	**<0.01**	85.5 ± 17.4	84.3 ± 20.0	0.704	77.1 ± 16.1	77.3 ± 14.5	0.963
BMI (kg/m^2^)	30.0 ± 6.0	27.1 ± 4.5	**<0.01**	30.4 ± 6.1	29.4 ± 5.9	0.307	27.3 ± 4.6	26.8 ± 4.4	0.490
Waist (cm)	100.4 ± 14.8	91.3 ± 13.2	**<0.01**	102.0 ± 13.7	97.8 ± 16.1	0.091	92.3 ± 12.8	89.4 ± 13.9	0.197
SBP (mmHg)	128.7 ± 19.9	124.3 ± 17.6	**0.043**	130.5 ± 20.1	125.8 ± 19.4	0.164	126.1 ± 17.9	120.8 ± 16.5	0.080
DBP (mmHg)	79.3 ± 9.7	77.4 ± 10.3	0.111	79.7 ± 9.7	78.5 ± 9.7	0.471	78.3 ± 10.7	75.7 ± 9.5	0.140
Hb (g/L)	145.8 ± 12.7	145.4 ± 13.0	0.764	147.1 ± 12.5	143.8 ± 12.8	0.123	145.8 ± 13.2	144.5 ± 12.8	0.547
Hct (L/L)	0.4 ± 0.0	0.4 ± 0.0	0.544	0.4 ± 0.0	0.4 ± 0.0	0.122	0.4 ± 0.0	0.4 ± 0.0	0.666
RCC^	4.8 ± 0.4	4.7 ± 0.4	0.105	4.8 ± 0.4	4.8 ± 0.4	0.271	4.7 ± 0.5	4.7 ± 0.4	0.577
RDW (%)	4.8 ± 6.0	4.0 ± 5.8	0.296	3.5 ± 5.6	6.8 ± 6.2	**<0.01**	3.1 ± 5.4	5.8 ± 6.3	**<0.01**
WCC^∗^	6.5 ± 2.1	6.0 ± 1.6	**0.016**	6.3 ± 1.7	6.9 ± 2.6	0.112	6.1 ± 1.5	5.7 ± 1.7	0.086
Lymph^∗^ (×10^12^/L8)	2.0 ± 0.5	1.8 ± 0.5	**0.020**	2.0 ± 0.6	2.0 ± 0.7	0.864	1.9 ± 0.6	1.8 ± 0.5	0.753
Mono^∗^	0.5 ± 0.1	0.4 ± 0.1	0.051	0.4 ± 0.2	0.5 ± 0.1	**0.044**	0.4 ± 0.2	0.4 ± 0.1	0.579
Eosin^∗^	0.2 ± 0.01	0.2 ± 0.01	0.735	0.2 ± 0.1	0.2 ± 0.1	0.824	0.2 ± 0.1	0.2 ± 0.1	0.401
Baso^∗^	0.04 ± 0.03	0.04 ± 0.03	0.523	0.04 ± 0.04	0.04 ± 0.05	0.962	0.04 ± 0.03	0.03 ± 0.03	0.114
Neutro^∗^	3.8 ± 1.2	3.5 ± 1.2	**0.039**	4.1 ± 1.9	3.6 ± 1.3	0.064	3.6 ± 1.2	3.2 ± 1.2	**0.032**
Platelets	246.4 ± 67.0	247.8 ± 61.7	0.849	239.2 ± 58.6	258.0 ± 77.9	0.096	244.4 ± 62.1	254.5 ± 61.0	0.348
MCV (fL)	88.8 ± 4.2	89.8 ± 3.9	**0.041**	89.0 ± 4.1	88.5 ± 4.3	0.448	89.7 ± 4.0	90.0 ± 3.9	0.699
TP (g/L)	74.7 ± 5.4	74.6 ± 3.8	0.833	75.0 ± 6.3	74.3 ± 3.5	0.460	74.8 ± 3.9	74.2 ± 3.5	0.413
Alb (g/L)	46.2 ± 3.4	45.7 ± 2.4	0.210	46.2 ± 3.8	46.1 ± 2.6	0.931	45.5 ± 2.6	46.3 ± 1.9	0.055
Bili (*μ*mol/L)	9.9 ± 5.5	10.3 ± 5.4	0.587	10.1 ± 5.4	9.7 ± 5.6	0.633	10.8 ± 6.0	9.2 ± 3.7	0.087
Gluc (mmol/L)	6.8 ± 2.4	5.2 ± 0.5	**<0.01**	6.9 ± 2.6	6.5 ± 2.0	0.223	5.2 ± 0.5	5.1 ± 0.4	0.223
Insulin (mU/L)	13.3 ± 9.3	7.7 ± 5.4	**<0.01**	13.7 ± 9.3	12.6 ± 9.3	0.516	8.1 ± 6.0	6.9 ± 4.2	0.175
CRP (mg/L)	3.3 ± 4.4	2.5 ± 3.2	0.065	2.9 ± 2.9	4.0 ± 6.2	0.173	2.6 ± 2.8	2.4 ± 3.7	0.770
Chol (mmol/L)	5.2 ± 1.2	5.5 ± 1.0	**0.033**	5.2 ± 1.1	5.2 ± 1.2	0.923	5.5 ± 0.9	5.4 ± 1.0	0.400
HDL (mmol/L)	1.3 ± 0.4	1.6 ± 0.5	**<0.01**	1.3 ± 0.4	1.3 ± 0.4	0.637	1.6 ± 0.5	1.6 ± 0.5	0.898
LDL (mmol/L)	3.1 ± 1.0	3.3 ± 0.9	0.066	3.1 ± 1.0	3.0 ± 0.9	0.545	3.3 ± 0.8	3.2 ± 0.9	0.624
Trig (mmol/L)	1.8 ± 1.1	1.4 ± 0.6	**<0.01**	1.8 ± 0.9	1.9 ± 1.2	0.331	1.4 ± 0.7	1.3 ± 0.5	0.230
ALP (U/L)	72.8 ± 21.7	67.9 ± 21.6	0.051	75.5 ± 22.9	68.5 ± 18.9	0.055	66.8 ± 19.1	70.0 ± 25.7	0.390
ALT (U/L)	17.9 ± 2 5.0	14.0 ± 7.3	0.069	20.1 ± 30.8	14.4 ± 8.9	0.178	14.2 ± 7.8	13.7 ± 6.4	0.736
Creat (*μ*mol/L)	77.4 ± 18.3	76.9 ± 17.4	0.883	81.1 ± 20.0	74.5 ± 16.6	0.199	77.7 ± 20.3	76.0 ± 14.6	0.766

Values are shown as mean ± standard deviation. T2DM: subjects with type 2 diabetes mellitus; *n*: number of subjects; +: subjects in which *T. gondii* IgG antibodies were detected; -: subjects in which *T. gondii* IgG antibodies were not detected; BMI: body mass index; cm: centimeter; kg: kilogram; kg/m^2^: kilogram force per square meter; SBP: systolic blood pressure; DBP: diastolic blood pressure; mmHg: millimeters of mercury; Hb: haemoglobin; Hct: haematocrit; RCC: red cell count; RDW: red cell distribution width; WCC: white cell count; MCV: mean cell volume; TP: total protein; Alb: albumin; Bili: bilirubin; Gluc: glucose; Chol: cholesterol; Trig: triglycerides; CRP: c-reactive protein; HDL: high-density lipoproteins; LDL: low-density lipoproteins; ALP: alkaline phosphatase; ALT: alanine transaminase; Creat: creatinine. Units are recorded as absolute counts ^∗^(×10^9^ cells/mL), or ^(×10^12^ cells/L).

## Data Availability

The datasets generated during the current study are available from the corresponding author on reasonable request. The pre-collected data analysed during this study are available from The Busselton Population Medical Research Institute but restrictions apply to the availability of these data, which were used under specific approval for the current study, and so are not publicly available.
